# The Lentiviral Vector Pseudotyped by Modified Rabies Glycoprotein Does Not Cause Reactive Gliosis and Neurodegeneration in Rat Hippocampus

**DOI:** 10.29252/.23.5.324

**Published:** 2019-09

**Authors:** Mostafa Farzaneh, Mohammad Sayyah, Hamid Reza Eshraghi, Negar Panahi, Hadi Mirzapourdelavar, Hamid Gholami Pourbadie

**Affiliations:** 1 Department of Pharmacology, Science and Research Branch, Islamic Azad University, Tehran, Iran;; 2 Department of Physiology and Pharmacology, Pasteur Institute of Iran, Tehran, Iran

**Keywords:** Astrocytes, CA3 region, Rat

## Abstract

**Background:**

A human immunodeficiency virus type 1 (HIV-1)-based lentiviral vector (LV) pseudotyped by a variant of rabies envelope glycoprotein, FUG-B2, has previously been prepared and used in transfection of hippocampal CA1 ("Cornu Ammonis" area 1) neurons. This study aimed to verify reactive gliosis and neuronal damage after injection of the vector into the rat hippocampus.

**Methods:**

HEK 293T cells were transfected with transfer (fck-Jaws-GFP-ER2), envelope (FUG-B2), and packaging (pMDLg/pRRE, pRSV-Rev) plasmids, and the vector was injected into CA1 of the rat hippocampus. After one week, transduction efficiency, and the number of neuronal and astroglial cells were determined in CA1 and CA3 by double staining of the brain slices.

**Results:**

Hippocampal cells were successfully transfected as 92.7% of CA1 and 95.8% of CA3 neuronal cells expressed GFP. The frequency of neuronal and astroglial cells in CA1 and CA3 of the vector-injected rats remained unchanged compared to those in the control and the saline-injected rats. Furthermore, no morphological change was found in hippocampal astrocytes and neuronal cells.

**Conclusion:**

The HIV-1-based LV pseudotyped by FUG-B2 is safe and does not cause neuroinflammation and neuronal loss once directly delivered into the rat hippocampus.

## INTRODUCTION

Lentiviral vectors (LV) are accomplished gene transfer vehicles capable of stable integrating and long-term expression of the transgene in non-dividing cells such as neurons. One of the highly promising LVs for treating central nervous system (CNS) disorders is the human immunodeficiency virus type 1 (HIV-1)-based LV pseudotyped by rabies envelope glycoprotein^[^^1^^]^. Delivery of this LV into the peripheral and CNS is accompanied by the successful transfer of the gene into the neurons distal to the injection site by retrograde axonal transport^[2,3]^. We have recently generated a HIV-1-based LV pseudotyped by a variant of rabies glycoprotein, fusion glycoprotein variant B2 (FUG-B2), with an acceptable titer^[^^4^^]^. The LV showed high gene transfer efficiency in the Schaffer collateral pathway in both postsynaptic neurons existing at the delivery sites CA1 and presynaptic neurons in CA3, by retrograde axonal transport^[4]^. Moreover, the transgene was highly expressed in both CA1 and CA3 pyramidal neurons for at least two months after vector injection^[^^4^^]^.

Rapid and stable integration in dividing and non-dividing cells as well as long-term and highly efficient transgene expression are the major functional features crucial in designing the LV system for experimental and clinical gene therapy studies. Yet, the potential immunological/inflammatory response is the essential concern in *in vivo* applications of LV systems^[^^5^^-^^7^^]^. Meanwhile, LV immunogenicity in CNS has inordinate importance due to the risk of neurotoxicity and neuronal loss induced by neuroinflammation. In follow-up to our previous work, the present study examined possible neuroinflammation and neurotoxicity of the LV pseudotyped by FUG-B2 in rat hippocampus. 

## MATERIALS AND METHODS

**Materials**

The monoclonal rabbit anti-green fluorescent protein (GFP), mouse anti-neuronal nuclear protein (NeuN), mouse monoclonal anti-glial fibrillary acidic protein (GFAP), and secondary Texas Red-X conjugated goat anti-mouse IgG antibodies were purchased from Merck, Millipore (USA). Penicillin-streptomycin was purchased from Gibco (USA). Fetal bovine serum (FBS), calcium phosphate, Dulbecco’s modified Eagle’s medium (DMEM), bovine serum albumin (BSA), Triton X-100, Trypsin, paraformaldehyde, secondary anti-rabbit fluorescein isothiocyanate-FITC antibody, and goat serum were procured from Sigma-Aldrich (USA). Human embryonic kidney cells (HEK 293T) were obtained from the Cell Bank of Pasteur Institute of Iran (IPI, Tehran). The third generation lentiviral packaging plasmids pMDLg/pRRE (Addgene plasmid # 12251) and pRSV-Rev (Addgene plasmid # 12253) were a gift from Didier Trono^[^^8^^]^. The insert DNA fck-Jaws-GFP-ER2 was a gift from Edward Boyden (Addgene plasmid # 65013)^[^^9^^]^. The envelope plasmid contained the cDNA encoding the modified rabies glycoprotein, FUG-B2, was kindly provided by Kazuto Kobayashi, Fukushima Medical University, Japan. 

**Animals**

Adult male Wistar rats (280–320 g, n = 7) were obtained from IPI. Rats were housed in standard polypropylene cages in a room under a 12:12 h light/dark cycle (07.00 AM to 07.00 PM) with controlled temperature (23 ± 2.0 °C). They were fed *ad libitum* with rodent's chow and had free access to drinking water. All experiments were conducted during light phase according to the guidelines of Institutional Animals Ethics Committee of IPI (Authorization code: IR.PII.REC.1394.48) and EU Directive 2010/63/EU in a way that minimized the number of animals required and their suffering.

**Lentiviral vector** **preparation**

HEK 293T cells were cultured in DMEM containing 10% FBS and penicillin-streptomycin of 100 units/ml with 5% CO_2_ at 37 °C for 24-48 hours. The cells were transfected with transfer (fck-Jaws-GFP-ER2, 13 μg), envelope (FUG-B2, 3.75 μg), and packaging (pMDLg/pRRE, 13 μg; pRSV/Rev, 3 μg) plasmids by the calcium-phosphate precipitation method. Twenty four hours after transfection, the medium was replaced with a fresh medium, and the cells were incubated for 24 h. The medium was then harvested and filtered through a 0.45-μm filter unit (Macherey-Nagel, Germany). Viral vector particles were pelleted by centrifugation at 50,000 ×g at 4 °C for 2 h and resuspended in phosphate-buffered saline (PBS)-BSA 1% solution and kept in -80 ºC. To titrate LV, 1 × 10^6 ^HEK 293T cells were seeded on 24-well plate with 1-ml medium per well. The cells were transduced 12 h thereafter with the serial dilutions of the vector. After four days, the number of GFP-positive cells in each dilution was counted by using the fluorescent microscope (Nikon, Japan). The vector titer (transducing units, TU) was determined using the following formula: 

Titer (TU/ml) = [GFP-positive cells × cells plated the first day (1 × 10^6^)]/dilution

**Delivery of the** **lentiviral vector** **into the rat hippocampus**

Two microliters of the LV stock (2 × 10^8^ TU/ml) was infused twice (with a 24-h interval between the injections) into the CA1 region (n = 3) of the right dorsal hippocampus according to the previously described method^[^^4^^]^. In the control group (n = 3), rats received physiologic saline (NaCl 0.9%, pH 7.4) instead of the LV. A positive control group was assigned to show reactive astrogliosis in the rat brain after neuronal damage. Traumatic brain injury (TBI) was induced in an anaesthetized rat by a controlled cortical impact (CCI) device according to the previously described method^[^^10^^]^. In brief, a 5-mm burr hole was drilled at left parieto-temporal cortex (coordinates; A, -4 mm from bregma; L, -4 mm from bregma). The bone was removed, and TBI was delivered by a CCI device (AmScien Instruments, Model AMS 201, USA) with 5 mm round tip, 4.5 mm/s velocity, 150 ms duration, and 2 mm depth of deformation. Then the dissected bone was brought back to its position on the skull and fixed with dental acrylic, and the skin was closed.

**Monitoring rats behavior after** **lentiviral vector** **injection**

Behavior of rats was monitored during the first-week after the injection of the LV and saline, or the induction of TBI. Any abnormal behavior such as sedation, agitation, and hyperactivity was recorded.

**Immunohistochemistry**

The brain of rats was dissected out seven days after infusion of the LV and saline, or the induction of TBI. Preparing the brain coronal sections and immunohistochemistry were performed according to the previously described method^[^^4^^]^. Eight-μm-thick coronal sections were made using a tissue slicer (Leica, Germany), processed and incubated with anti-NeuN antibody, 1:700. Another series of the sections were incubated with anti-GFAP antibody, 1:400. The sections were then incubated with Texas Red-X conjugated goat anti-mouse IgG secondary antibody, 1:1000. After washing, the sections were cover-slipped with 90% glycerol mounting buffer and visualized in the dark place with fluorescent microscope (Nikon) equipped with fluorescence filter Set for Texas Red dye For double staining, and brain sections were incubated with anti-GFP (1:1000) and anti-NeuN antibody (1:700). The sections were then incubated with either Texas Red-X conjugated goat anti-mouse IgG secondary antibody (1:1000) or FITC-conjugated goat anti-rabbit IgG, 1:500. After washing, the sections were cover-slipped with 90% glycerol mounting buffer and visualized in a dark place with a fluorescent microscope (Nikon) equipped with specific ﬁlter sets for ﬂuorescein Texas Red-X and FITC. Digital photographs were taken by a digital camera connected to the microscope using ×20 objective lens. The number of GFP, GFAP, NeuN, and GFP/NeuN positive cells were counted in CA1 and CA3 regions of every 10 sections (400 × 350 µm^2^) by computer-assisted imaging program (Image J, version 1.8).

**Statistical analysis**

Cell numbers were expressed as the mean ± SEM. Statistical analyses were performed using Prism version 6 (GraphPad Software, USA). Unpaired student's *t*-test was used to analyze the data of control and LV-injected groups. *p* < 0.05 was considered statistically significant.

## RESULTS

**Fig. 1:**
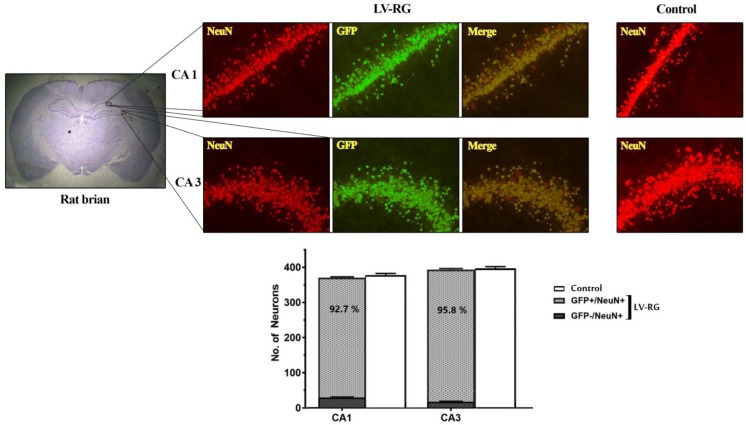
Schematic representation and frequency of green fluorescent protein (GFP)-expressed neurons in CA1 and CA3 of the rat hippocampus one week after injection of the lentiviral vector into CA1. Upper part, left: a coronal section of the rat brain stained by hematoxylin and eosin. Upper part, LV-RG: double-staining of the hippocampal cells of the vector-injected rats by anti-GFP and anti-NeuN monoclonal antibodies. Upper part, control: staining of the hippocampal cells of the saline-injected rats by anti-neuronal Nuclear protein (NeuN) monoclonal antibody. Lower part: frequency of neurons in CA1 and CA3 regions of rat hippocampus, one week after delivery of the lentivector or saline into CA1. No significant difference was found in the frequency of NeuN-positive cells between control and the vector-injected groups in both regions. LV-RG: the HIV-1-based lentiviral vector pseudotyped by the modified rabies glycoprotein, FUG-B2. Scale bar: 100 µm. Magnification: 20  , 400 × 350 µm^2^

The titer of the lentivector was found 2 × 10^8^ TU/ml. No abnormal behavior was observed in rats during one-week period after the injection of the LV and saline or the induction of TBI. Double staining of the hippocampal cells by GFP/NeuN antibodies showed that the majority of CA1 and CA3 neurons were GFP-positive (Fig. 1). Cell counting revealed that more than 90% of both CA1 and CA3 neurons was transduced.

**Fig. 2:**
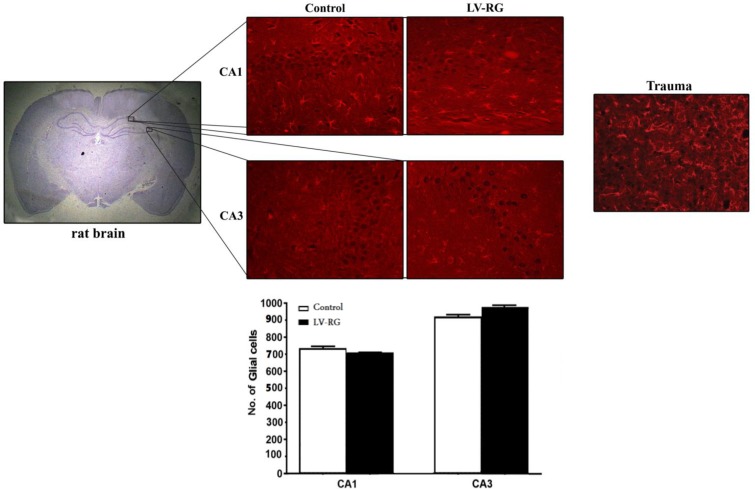
Schematic representation and frequency of glial cells in CA1 and CA3 of the rat hippocampus one week after injection of the lentiviral vector into CA1. Upper part, left: a coronal section of the rat brain stained by hematoxylin and eosin. Upper part, middle: immunostaining of the hippocampal cells of the vector (LV-RG)-or saline (control)-injected rats by anti-glial fibrillary acidic protein (GFAP) monoclonal antibody. Upper part, trauma: massive astrogliosis in the damaged brain cortex of the traumatic rat immunostained by anti-GFAP monoclonal antibody. Lower part: Frequency of glial cells in CA1 and CA3 regions of rat hippocampus, one week after delivery of the lentivector or saline into CA1. No significant difference was found in the frequency of GFAP-positive cells between the control and vector-injected groups in both regions. LV-RG: the HIV-1-based lentiviral vector pseudotyped by the modified rabies glycoprotein, FUG-B2. Scale bar: 100 µm. Magnification: 20 , 400× 350 µm^2^

Neither morphology change nor damage was observed in the transfected neurons. Figure 2 represents GFAP-expressing cells in the traumatic brain and CA1/CA3 of the LV-injected rats. Major astrogliosis was observed in the margin of the traumatic area in the subcortical regions. GFAP-positive cells were visualized clearly in both CA1 and CA3 regions. However, the number of astrocytes and their morphology in CA1 and CA3 of the saline-injected rats had no significant difference from those of the LV-injected rats.

## DISCUSSION

We showed that the HIV-1-based LV pseudotyped by FUG-B2 does not cause astrogliosis and neuronal loss in both the injection site CA1 and target area CA3, one week after injection to rat brain. Inflammation is an important component of the multicellular response to a wide range of mild to severe CNS damage that can markedly influence the balance between tissue loss and preservation. There is a plenty of clinical and experimental evidence indicating that astrocytes are critical regulators of CNS inflammation^[11]^. Astrocytes are a sub-type of glial cells in CNS and react to CNS challenges by hypertrophy, proliferation, and overexpression of their GFAP near the site of injury. The increased GFAP immunoreactivity is the key index of gliosis and correlates with neural damage. By sensing signals of damage or injury, astrocytes and microglia undertake a gradual activation process leading to morphological changes and secretion of pro-inflammatory elements that eventually trigger neuronal death^[^^12^^]^. Hence, we examined astrogliosis as the early sensor of CNS damage, to detect any potential neuropathologic effect of our LV. We did not find any astrogliosis in the site of LV delivery, CA1, and also in the target region CA3 where the vector reached by retrograde axonal transport.

We have recently reported that the HIV-1-based LV pseudotyped by FUG-B2 is able to transduce more than 90% of hippocampal neurons one week after intra-hippocampal injection^[^^4^^]^. Meanwhile, the process of gliosis, considering astrogliosis as the final component of this process, takes place over several days^[^^13^^]^. Therefore, the one-week period after LV injection seems to be an appropriate time to reveal any potential damage to CNS.

It has been reported that pseudotyping equine infectious anemia virus-based vector by the envelope G protein of the wild-type rabies, Evelyn-Rokitnicki-Abelseth, induces neuroinflammatory response after delivery to the rat brain^[^^2^^]^. The envelope of our LV is FUG-B2. FUG-B is composed of the extracellular and transmembrane domains of the glycoprotein derived from the rabies virus, challenged virus standard strain, and the cytoplasmic domain of glycoprotein of the vesicular stomatitis virus^[^^14^^]^. FuG-B2 is a variant of FUG-B cDNA in which a part containing the domains of the rabies virus glycoprotein is exchanged with the corresponding part derived from the rabies virus, Pasteur virus strain^[^^14^^]^. Based on a previous report, injection of the HIV-1-based LV pseudotyped with FUG-B2 to the tongue muscle of mice has no inflammatory sign at the injection site^[^^3^^]^. We report here for the first time that the HIV-1-based LV pseudotyped with FUG-B2 is safe and does not induce neuroinflammation and neuronal loss at the injection as well as target regions in the rat hippocampus. 

Opsins are light-sensitive proteins used in genetic engineering enabling neurons to transiently be excited or inhibited in response to light. Jaws is derived from Haloarcula (Halobacterium) salinarum (strain Shark), which enables neurons to be inhibited in response to red light both *in vitro* and *in vivo*^[^^9^^]^. The transfer plasmid used in LV construct in the present study is fck-Jaws-GFP-ER2. Chuong *et al.*^[^^9^^]^ have shown that the activity of Jaws-GFP-ER2-expressing neurons in brain of awake mice is inhibited in response to red light. However, they did not examine possible changes in the morphology of Jaws-GFP-ER2-expressing neurons and glial inflammation^[^^9^^]^. The LV system used in our study is different from that applied in Chuong *et al.*'s^[^^9^^]^ study. Yet, the transgene used in both studies was the same and we did not find any gliosis and damage in the Jaws-GFP-ER2-expressing neurons in the rat hippocampus. 

In conclusion, the HIV-1-based LV pseudotyped by FUG-B2, and the transgene, Jaws, did not cause inflammation and neuronal injury, in the rat hippocampus. The efficient gene delivery of this LV into the hippocampus along with adequate neuronal safety may open a path toward the inhibition of neurological disorders arising from hippocampal hyperexcitability. 
